# Fetuin‐A, inter‐α‐trypsin inhibitor, glutamic acid and ChoE (18:0) are key biomarkers in a panel distinguishing mild from critical coronavirus disease 2019 outcomes

**DOI:** 10.1002/ctm2.704

**Published:** 2022-01-24

**Authors:** Laia Reverté, Elena Yeregui, Montserrat Olona, Alicia Gutiérrez‐Valencia, Maria José Buzón, Anna Martí, Frederic Gómez‐Bertomeu, Teresa Auguet, Luis F. López‐Cortés, Joaquin Burgos, Clara Benavent‐Bofill, Carme Boqué, Graciano García‐Pardo, Ezequiel Ruiz‐Mateos, Maria Teresa Mestre, Francesc Vidal, Consuelo Viladés, Joaquim Peraire, Anna Rull, COVIDOMICS Study Group

**Affiliations:** ^1^ Hospital Universitari de Tarragona Joan XXIII (HJ23) Tarragona Spain; ^2^ Institut Investigació Sanitària Pere Virgili (IISPV) Tarragona Spain; ^3^ CIBER Enfermedades Infecciosas (CIBERINFEC) Instituto de Salud Carlos III Madrid Spain; ^4^ Universitat Rovira i Virgili (URV) Tarragona Spain; ^5^ Unit of Infectious Diseases Microbiology and Preventive Medicine Virgen del Rocío University Hospital Seville Spain; ^6^ Institute of Biomedicine of Seville (IBiS) Virgen del Rocío University Hospital/CSIC/University of Seville Seville Spain; ^7^ Department of Infectious Disease Hospital Universitari Vall d'Hebron Institut de Recerca (VHIR) Universitat Autònoma de Barcelona Barcelona Spain

Dear Editor,

The mechanistic pathways leading to immune dysregulation and complications driven by uncontrolled severe acute respiratory syndrome coronavirus‐2 (SARS‐CoV‐2) infection remain major challenges.^1^, ^2^ Hence, a detailed analysis of the proteome, metabolome and lipidome profile of coronavirus disease 2019 (COVID‐19) patients showing different severity grades might shed light on the disease pathophysiology and unveil new predictive biomarkers to promptly ascertain patient's outcomes.

Our COVID‐19 study cohort included 273 SARS‐CoV‐2 infected individuals recruited during the first wave (March–April 2020) in three different hospitals and grouped by the disease severity following the medical inclusion criteria^3^ in mild, severe or critical (Figure 1A), from whom demographic, preexisting clinical conditions and COVID‐19 treatments are summarized in Table S1. The greatest significant differences were observed between mild and critically ill patients. These findings indicated that older individuals with comorbidities such as hypertension, obesity, diabetes and cardiovascular disorders, mostly presenting dyspnea (Figure 1B), may be at higher risk of suffering from severe respiratory distress with subsequent oxygen and drug requirements and, eventually, died. Similarly, the serum biochemical composition analysis revealed a well‐differentiated blood pattern previously defined for critically ill patients (Figure S1).

**FIGURE 1:**
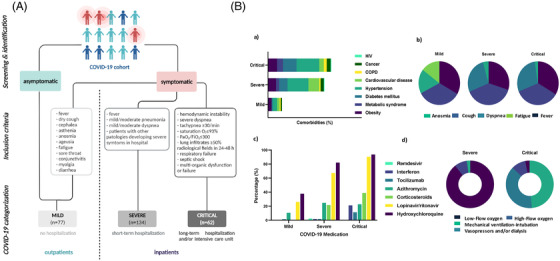
Study design and clinical characterization of the study cohort. (A) Flowchart of the clinical strategy followed to categorize patients of the coronavirus disease‐2019 (COVID‐19) study cohort. (B) Incidence of comorbidities (a), COVID‐19 symptoms (b), medication (c), and oxygen & intensive care (d), grouped by disease severity as mild, severe and critical patients. The size of bars (a, c) and circular (b, d) portions is proportional to the percentage of the corresponding comorbidity, symptom, medication or treatment. While patients with mild disease presented mostly anosmia, were treated with antibiotics and did not require oxygen supply, the incidence of dyspnea was significantly higher in the severe and critical groups, many of the latter requiring corticosteroids, hydroxychloroquine, lopinavir/ritonavir. Low‐flow oxygen therapies were mainly necessary for severe patients, some of who required high‐flow oxygen administration and, a high proportion of critical patients were intubated and required vasopressor administration or dialysis. Please note that COVID‐19‐related medication (b) was dispensed after blood sample collection, so that is assumed the subsequent analysis are not biased due to exposure to medication at the time of blood collection

In light of the promising results already provided by omic technologies in the search for predictive biomarkers of COVID‐19 severity,^4^, ^5^ we conducted a nontargeted multi‐omic, including proteomic, metabolomic and lipidomic analyses, in the serum from patients of the COVID‐19 study cohort. The proteomics analysis identified 65 proteins with a significant increase or decrease in abundance according to the disease severity (Figure 2A), which resulted to be highly interconnected (Figure 2B). Hence, the complement and coagulation cascades were markedly the most significantly enriched pathways related to COVID‐19 severity (Figure 2C). Other protein‐coding genes such as carboxypeptidases, protease inhibitors, acute phase proteins, extracellular matrix stabilizers and antimicrobial enzymes, were also significantly up‐regulated in severely and critically ill patients. These results showed the essential contribution of these proteins in the coagulopathy phenomenon and hyperinflammatory state that subsequently enhances SARS‐CoV‐2 endocytosis and infectivity and promotes secondary bacterial infections, previously described as aggravators of severe and critical COVID‐19 cases.^6^ Proteins with reduced abundance in critically ill patients with COVID‐19 were mostly associated with lipid transport (apolipoproteins), which dysfunction seems to increase SARS‐CoV‐2 infectivity in patients with COVID‐19.^7^ For the first time, fetuin‐A (AHSG) and inter‐α‐trypsin inhibitor 3 (ITIH3) were determined as the most accurate biomarkers (random forest) of the critical clinical progression of COVID‐19 (Figure 2D).

**FIGURE 2:**
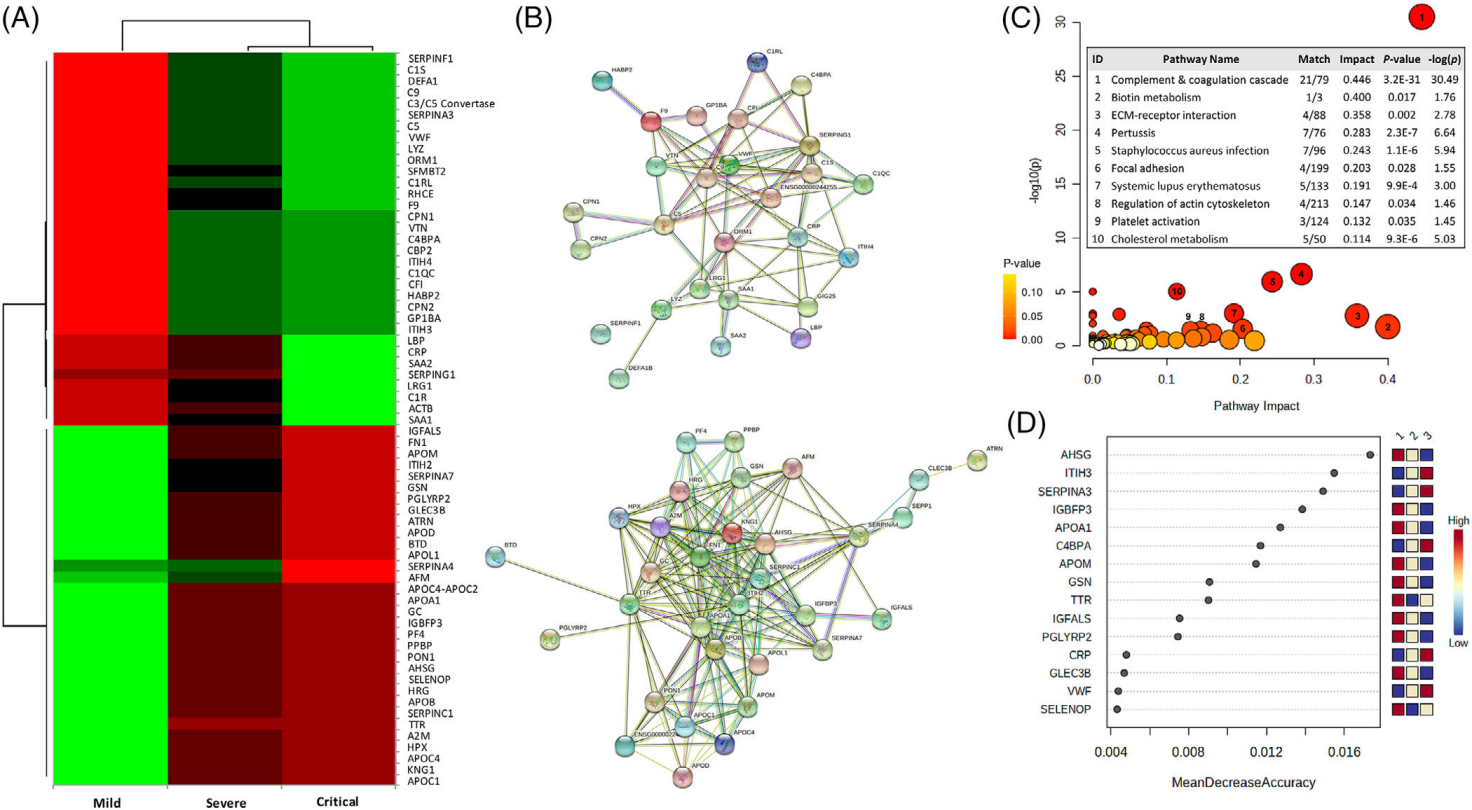
Serum proteomics profile of coronavirus disease‐2019 (COVID‐19) study cohort. (A) Heatmap showing significant proteins increasing or decreasing in accordance with disease severity. Columns correspond to the degree of disease severity: mild (left), severe (centre) and critical (right) groups. Mean values for each compound in each coronavirus disease‐2019 (COVID‐19) group (columns) are colour‐coded based on relative abundance, low (red) & high (green). Among the 65 significant proteins, 33 increased and 32 decreased with disease severity. (B) Up‐(upper) and down‐(bottom) regulated protein networks sorted by gene‐name showing a tight interconnection within the up‐ and down‐regulated proteins. (C) Kyoto Encyclopedia of Genes and Genomes (KEGG) enrichment analysis sorted by pathway impact and ‐log10 (p). The interconnected up‐ and down‐regulated genes were enriched in 77 pathways, and the 10 relevant pathways whose impact values were greater than 0.1 (*p* < .05) were further considered. The bubble diagram shows matched pathways according to the p‐values and pathway impact values. The size of bubbles shows pathway impact value and the colour denote the level of significance by means of *p*‐values. Numbers in circles correspond to the significantly enriched pathways ordered from the highest to the lowest pathway impact value. The number of matched proteins, impact value and *p*‐value corresponding to each pathway are indicated on the inserted table. (D) Random forest analysis showing the 15 protein‐encoding genes ranked by classification accuracy to distinguish between a mild and critical group of patients. Squares on the right represent the COVID‐19 (1 = mild, 2 = severe and 3 = critical) and the colours depict the accuracy power (red and blue for high and low accuracy, respectively)

The metabolomic and lipidomic analyses revealed 34 metabolites and 28 lipids that were significantly increased or decreased in relation to severity (Figure 3A). Interestingly, many of the altered metabolites were amino acids and sugars involved in central carbon metabolism. In line with previous reports,^8^, ^9^ critically ill patients showed a significant increase in glucose and glutamic acid (GA) levels but a reduction in glutamine, citrate and uric acid levels, suggesting mitochondrial dysfunction, an enhanced glutaminolysis and a shift from anaerobic to aerobic glycolysis (Warburg effect). Accordingly, D‐glutamine and D‐glutamate metabolism were the most significantly enriched pathways (Figure 3B, left panel), and were significantly related to seizures disorders, anoxia, heart failure, diabetes, obesity and inflammatory diseases (Figure 3B, right panel). Lipid levels that increased with severity were mainly triglycerides (TGs) and diacylglycerols, and those that decreased were predominantly sphingomyelins (SMs), cholesteryl esters (ChoEs) and lysophosphatidylcholines. Lipoproteins rich in TGs may trigger dysfunction in innate immunity and impair the defence mechanism against COVID‐19^10^ and a reduced abundance of SMs and ChoEs may interfere in signal transduction and in key immune and cellular processes. Among them, GA and ChoE (18:0) resulted in the most powerful (random forest) predictive biomarkers for COVID‐19 evolution (Figure 3C), confirmed by the prognosis accuracy determined by the receiver operating characteristic (ROC) analysis (Figure S2A–C, respectively). The highest accuracy was attained when combining both compounds in the distinction of mild from critical illnesses (Figure 2SD). To provide insights into the biological pathways related to the pathophysiology of the disease, we study the linkage and co‐regulation between the distinct classes of biomolecules by integrating the most significant demographical and clinical data (Table S1 and Figure S1) and the top omic molecules determined above (Figure S3A,B) in Spearman correlation matrix analyses (Figure 4A1–3). Despite all three groups showing a similar association pattern for most of the variables analyzed, patients with mild illness (Figure 4A1) significantly differed from those of the severe and critical groups (Figure 4A2,3, respectively). In brief, significant correlations were obtained across the omic data, which were more intense between lipidomics than within the protein‐encoding genes, and nearly negligible through metabolomics. The predictive power of the selected omics biomolecules as biomarkers for the severe disease was subsequently demonstrated by the high accuracy, sensitivity and specificity obtained by combining the four molecules in the ROC analysis (Figure 4B) to effectively distinguish critical COVID‐19 patients from patients with mild disease (area under the curve [AUC] = 0.994). To precisely predict whether a patient will progress from severe to a life‐threatening disease, not only the four but all the top‐omic selected biomarkers need to be integrated into the ROC analysis (AUC = 0.811; Figure 4C).

**FIGURE 3:**
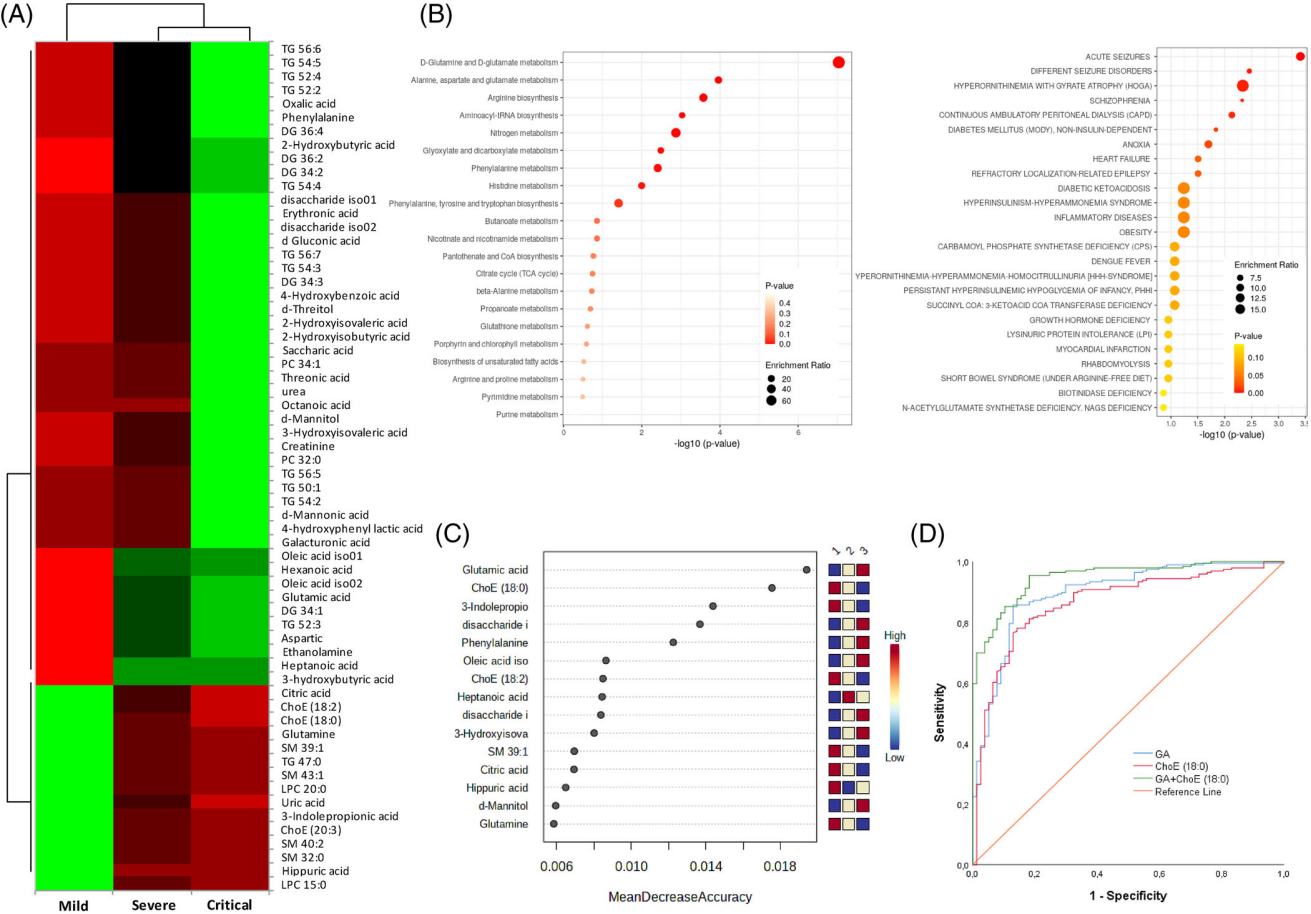
Metabolomic and lipidomic profile of coronavirus disease‐2019 (COVID‐19) patients grouped by disease severity. (A) Heatmap plots significant relative abundance of metabolites & lipids increasing or decreasing in accordance with disease severity. Significant differences (*p*‐values < .05) were determined by ANOVA test followed by post‐hoc Bonferroni correction for mean relative abundance between mild, severe and critical COVID‐19 groups of patients. Columns correspond to the degree of disease severity: mild (left), severe (centre) and critical (right) groups. Mean values for each compound in each COVID‐19 group (columns) are colour‐coded based on relative abundance, low (red) & high (green). (B) Metabolomic and lipidomic Kyoto Encyclopedia of Genes and Genomes (KEGG) enrichment analysis and related blood diseases in COVID‐19 patient cohort. Functional metabolic enrichment pathway of 15 metabolites selected from random forest modelling (Fisher's exact test. *p* < .05) (left), and the corresponding enriched blood pathway diseases (right). (C) Random forest modelling of significant metabolites and lipids with the highest classification accuracy. Right‐legend indicates the capacity of the compounds to differentiate groups of severity, blue (low) and red (high)

**FIGURE 4:**
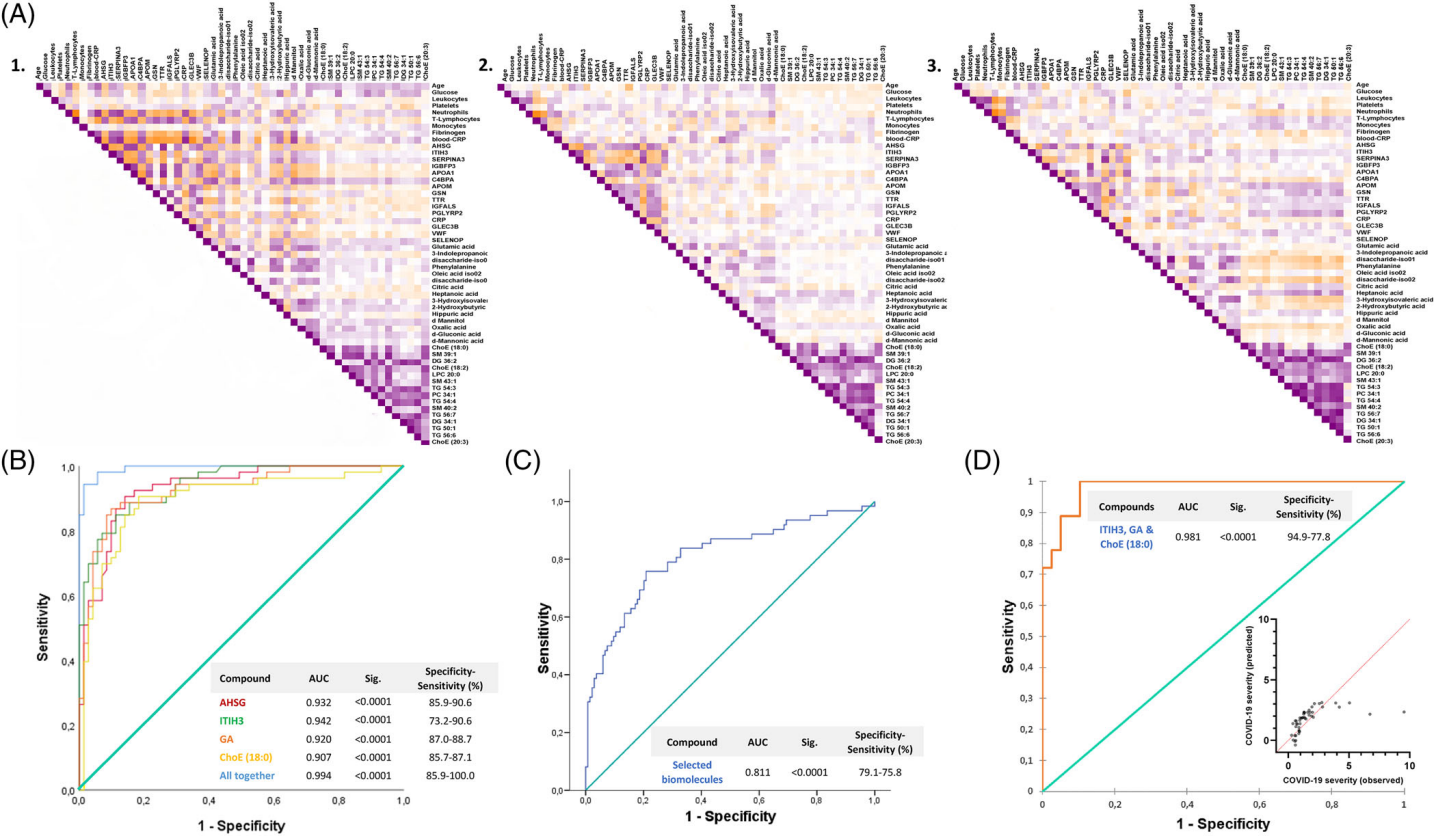
Evaluation of potential biomarkers to be a part of a panel to distinguish coronavirus disease‐2019 (COVID‐19) outcomes. (A) Heatmap showing the Spearman correlation coefficient of pairwise comparison between demographic, clinical and most enriched proteomic, metabolomic and lipidomic biomolecules determined in the blood of patients with mild (1), severe (2) and critical (3) COVID‐19. Spearman matrices are colour‐coded (‐1:1, orange:purple through white) and correlations with *p*‐values < .05 were considered statistically significant. (B) Receiver operating characteristic (ROC) curves analysis for the predictive power of top selected protein‐encoding genes, lipids and metabolites in random forest analysis to differentiate patients with mild from those with a critical illness. (C) ROC curve analysis for the most enriched proteomic, metabolomic and lipidomic biomolecules to differentiate severe from critically‐ill patients with COVID‐19. (D) Binary logistic regression modelling analysis testing the accuracy of the four selected biomarkers to differentiate mild from critically ill patients with COVID‐19 in a randomly selected set of patients

Taking a step further, the inclusion of AHSG, ITIH, GA and ChoE (18:0) in a predictive biomarker panel for COVID‐19 severity was validated in a randomly selected subset of patients. The regression modelling analysis confirmed the usefulness (classification accuracy >90%) of the biomarker panel in distinguishing mild to critical COVID‐19 outcomes (Figure 4D). Once more, all these findings highlighted the complex interactions between certain biological processes and the most serious complications arising from SARS‐CoV‐2 infections and revealed their potential as predictive biomarkers of disease severity.

Limitations are the small sample size to perform subgroup analyses and the lack of a non‐infected SARS‐CoV‐2 group of subjects. However, this study was conducted in a representative symptomatic well‐characterized Spanish cohort to determine predictive biomarkers of COVID‐19 severity.

In conclusion, the multi‐omic analysis identified new specific molecules related to complement and coagulation cascades, platelet activation, cell adhesion, acute inflammation, energy production (Krebs cycle and Warburg effect), amino acid catabolism and lipid transport as fingerprints of the acute disease. A novel biomarker panel consisting of AHSG, ITIH3, GA and ChoE (18:0) was proposed for the accurate differentiation of mild from critical COVID‐19 outcomes.

## FUNDING INFORMATION

This work has been developed in the framework of the COVIDOMICS’ project supported by Direcció General de Recerca i Innovació en Salut (DGRIS), Departament de Salut, Generalitat de Catalunya (PoC‐6‐17 and PoC1‐5). The research has also been funded by the Programa de Suport als Grups de Recerca AGAUR (2017SGR948), the SPANISH AIDS Research Network [RD16/0025/0006, RD16/0025/0007 and RD16/0025/0020]‐ISCIII‐FEDER (Spain), the Centro de Investigación Biomédica en Red de Enfermedades Infecciosas‐ISCIII [CB21/13/00020], Madrid, Spain and Consejeria de Transformacion Economica, Industria, Conocimiento y Universidades Junta de Andalucía (research Project CV20‐85418). Elena Yeregui was supported by the Instituto de Salud Carlos III (ISCIII) under grant agreement “FI20/00118″ through the programme “Contratos Predoctorales de Formación en Investigación en Salud”. Laia Reverté was supported by the Instituto de Salud Carlos III (ISCIII) under grant agreement “CD20/00105″ through the programme “Contratos Sara Borrell”. Francesc Vidal was supported by grants from the Programa de Intensificación de Investigadores (INT20/00031)‐ISCIII and by “Premi a la Trajectòria Investigadora dels Hospitals de l'ICS 2018″. Anna Rull was supported by a grant from IISPV through the project “2019/IISPV/05″ (Boosting Young Talent), by GeSIDA through the “III Premio para Jóvenes Investigadores 2019″ and by the Instituto de Salud Carlos III (ISCIII) under grant agreement “CP19/00146″ through the Miguel Servet Program. Maria José Buzón was supported by the Miguel Servet Program (CP17/00179). Ezequiel Ruiz‐Mateos was supported by the Spanish Research Council (CSIC). Alicia Gutiérrez‐Valencia was supported by the Instituto de Salud Carlos III, cofinanced by the European Development Regional Fund (“A way to achieve Europe”), Subprograma Miguel Servet (grant CP19/00159). This project was also funded by a donation from the city Council of Perafort (to Teresa Auguet).

## CONFLICT OF INTEREST

The authors declare that they have no conflict of interest.

## Supporting information

Supporting Information

## References

[1] WHO . COVID‐19 therapeutic trial synopsis. World Health Organization. 2020;1‐9

[2] Domingo P , Mur I , Pomar V , et al. The four horsemen of a viral apocalypse: the pathogenesis of SARS‐CoV‐2 infection (COVID‐19). EBioMedicine. 2020;58:102887. 10.1016/J.EBIOM.2020.102887. 32736307 PMC7387269

[3] Wang GQ , Zhao L , Wang X , Jiao YM , Wang FS . Diagnosis and treatment protocol for COVID‐19 patients (tentative 8th edition). Infect Dis Immun. 2021;1:8‐16. 10.1097/01.id9.0000733564.21786.b0. PMC8057316 38630124

[4] Wang X , Xu G , Liu X , et al. Multiomics: unraveling the panoramic landscapes of SARS‐CoV‐2 infection. Cell Mol Immunol. 2021;18:2313‐2324. 34471261 10.1038/s41423-021-00754-0 PMC8408367

[5] McArdle A , Washington KE , Chazarin Orgel B , et al. Discovery proteomicsfor COVID‐19: where we are now. J Proteome Res. 2021;20:4627. 10.1021/ACS.JPROTEOME.1C00475. 34550702 PMC8482317

[6] Zhou F , Yu T , Du R , et al. Clinical course and risk factors for mortality of adult inpatients with COVID‐19 in Wuhan, China: a retrospective cohort study. Lancet. 2020;395:1054‐1062. 10.1016/S0140-6736(20)30566-3. 32171076 PMC7270627

[7] Wang H , Yuan Z , Pavel MA , et al. The role of high cholesterol in age‐related COVID19 lethality. BioRxiv. 2020. 10.1101/2020.05.09.086249.

[8] Wu D , Shu T , Yang X , et al. Plasma metabolomic and lipidomic alterations associated with COVID‐19. Nat Sci Rev. 2020;7:1157‐1168. 10.1093/NSR/NWAA086. PMC7197563 34676128

[9] Bharadwaj S , Singh M , Kirtipal N , Kang SG . SARS‐CoV‐2 and Glutamine: SARS‐CoV‐2 triggered pathogenesis via metabolic reprograming of glutamine in host cells. Front Mol Biosci. 2021;7. 10.3389/fmolb.2020.627842. PMC7873863 33585567

[10] McKechnie JL , Blish CA . The innate immune system: fighting on the front lines or fanning the flames of COVID‐19? Cell Host Microbe. 2020;27:863‐869. 32464098 10.1016/j.chom.2020.05.009 PMC7237895

