# Supplementary material for: Fetuin‐A, inter‐α‐trypsin inhibitor, glutamic acid and ChoE (18:0) are key biomarkers in a panel distinguishing mild from critical coronavirus disease 2019 outcomes
Source: Clin Transl Med. 2022 Jan 24;12(1):e704. doi: 10.1002/ctm2.704 (PMC8787095; doi:10.1002/ctm2.704)
Supplement: Supplementary file 1 — Supporting Information [file CTM2-12-e704-s001.docx]

**Supporting information**

**Fetuin-A, inter-α-trypsin inhibitor, glutamic acid and ChoE (18:0) are key biomarkers in a panel distinguishing mild from critical COVID-19 outcomes**

***Letter-to-Editor with previous submission number CTM2-2021-10-2185***

*Running title: Critical COVID-19 predictive biomarkers*

Laia Reverté^1,2,3,#^, Elena Yeregui^1,2,#^, Montserrat Olona^1,2,3,4^, Alicia Gutiérrez-Valencia^5,6^, Maria José Buzón^7^, Anna Marti^1,2^, Frederic Gómez-Bertomeu^1,4^, Teresa Auguet^1,2,4^, Luis F. López-Cortés^5,6^, Joaquin Burgos^7^, Clara Benavent-Bofill^1^, Carme Boqué^1,4^, Graciano García-Pardo^1,2,4^, Ezequiel Ruiz-Mateos^5,6^, Maria Teresa Mestre^1^, Francesc Vidal^1,2,3,4,*^, Consuelo Viladés^1,2,3,4^, Joaquim Peraire^1,2,3,4,φ^, Anna Rull^1,2,3,4,8,φ,*^ and COVIDOMICS Study Group^¥^

^1^ Hospital Universitari de Tarragona Joan XXIII (HJ23), Tarragona, Spain

^2^ Institut Investigació Sanitària Pere Virgili (IISPV), Tarragona, Spain

^3^ CIBER Enfermedades Infecciosas (CIBERINFEC), Instituto de Salud Carlos III, Madrid, Spain

^4^ Universitat Rovira i Virgili (URV), Tarragona, Spain

^5^ Unit of Infectious Diseases, Microbiology, and Preventive Medicine, Virgen del Rocío University Hospital, Seville, Spain

^6^ Institute of Biomedicine of Seville (IBiS), Virgen del Rocío University Hospital/CSIC/University of Seville, Seville, Spain

^7^ Infectious Disease Department, Hospital Universitari Vall d'Hebron, Institut de Recerca (VHIR), Universitat Autònoma de Barcelona, Barcelona, Spain

^8^ Lead contact

# These authors contributed equally to this paper, and should be considered as primary co-authors.

^φ^ These authors contributed equally to this paper, and should both be considered as senior co-authors.

* Correspondence: Francesc Vidal ([fvidalmarsal.hj23.ics@gencat.cat](mailto:fvidalmarsal.hj23.ics@gencat.cat)) and Anna Rull ([anna.rull@iispv.cat](mailto:anna.rull@iispv.cat))

Along with a potent viral replication capacity and fast spread, COVID-19 is a complex and highly variable disease with a broad range of clinical presentations, ranging from asymptomatic to mild, moderate, severe and fatal outcomes. In the initial course of the disease, infected individuals remain asymptomatic for days, a period that is followed by flu-like symptoms in symptomatic patients and culminates in a complex pattern including a strong inflammatory response, cardiovascular disorders, lung dysfunction and hypercoagulation in critical patients (Baden and Rubin, 2020; Yang et al., 2020) who need immediate access to specialized intensive care units (ICUs). In fact, critically ill patients with COVID-19 are often not able to restore lung perfusion due to thromboembolic complications and die [3]. Currently, there is no clear association between having certain risk factors and increased death rate and, the reasons of why some SARS-CoV-2-infected patients are able to fight the infection but others require hospitalization, and among them, approximately 10% die remains unknown. Hence, early diagnostic tools and predictive biomarkers must be developed to promptly identify patients more prone to evolve to life-threatening disease [4]. Omics datasets and proteomic signatures have emerged as robust and powerful tools able to provide a better understanding of immunopathogenesis and the host immune response to SARS-CoV-2 (Rana et al., 2020), as well as to identify potential drug targets. Along with proteomics, lipidomics and metabolomics technologies have been used to study the biological factors contributing to a worse prognosis of patients with COVID-19 [7]. Despite the substantial advances in the clinical management of the pandemic by the research community, the mechanistic pathways leading to immune dysregulation remain poorly understood. The detection of alterations in human proteins, metabolites and lipids will serve as indicators of pathophysiological changes triggered by SARS-CoV-2 infection and might extend the knowledge of COVID-19. For this purpose, complete characterization of plasma samples from large cohorts of patients with COVID-19 presenting the whole spectrum of disease severities becomes crucial. Accordingly, we propose a nontargeted multiomics approach to study the serum profile of 273 patients with mild to critical COVID-19 in the acute phase of the infection. Thus, the main objective is to improve our current knowledge of the pathophysiology and to identify key molecules involved in the mechanistic pathways of the disease for further exploitation as potential therapeutic targets.

**MATERIALS AND METHODS**

**Study design and classification criteria**

Patients enrolled in the present study accessed the hospital through different routes: home, ancient care homes, other hospitals or primary health care centers, or medical staff that agrees to participate in the study. The COVID-19 patient cohort comprises a total of 273 patients with SARS-CoV-2 infection confirmed by Polymerase chain reaction (PCR) within the first 21 days of the infection. According to the inclusion criteria described in “Diagnosis and Treatment Protocol for COVID-19 Patients (version 8 trial)”[8], COVID-19 patients were categorized into 3 groups of severity and distributed as follows: mild (n=77), severe (n=134) and critical (n=62) (Fig. 1A). All information was collected and stored in a database specially designed for this purpose. The aforementioned database contains data regarding the hospitalization, such as the symptoms presented at the time of admission, the radiological findings, the pneumonia degree, the oxygen therapy required, the medical treatment received, as well as demographic data and previous diseases of interest.

**Samples recruitment**

The sampling protocol performed included clinical evaluation, blood cell count, and standard biochemical parameters at inclusion (baseline). Serum samples were stored at -80ºC at BioBank - Institut d’Investigació Sanitària Pere Virgili (IISPV) facilities until used for omics analysis.

**Ethics**

Protocols were carried out in accordance with the recommendations of the Ethical and Scientific Committees from each participating institution and were approved by the Committee for Ethical Clinical Research following the rules of Good Clinical Practice from the IISPV (079/2020, CEIm IISPV) and from the Vall d´Hebron Hospital (PR(AG)192/2020). The CEIm IISPV is an independent committee, made up of health and non-health professionals, which supervises the correct compliance of the ethical principles governing clinical trials and research projects that are carried out in our environment, specifically in its methodology, ethics and laws. All subjects or their relatives gave written informed consent in accordance with the Declaration of Helsinki.

**Serum analysis by multi-omic technology**

***Proteomic analysis***

Before proteomic analysis, the depletion of the seven most abundant plasma proteins (Albumin, IgG, antitrypsin, IgA, transferrin, haptoglobin and fibrinogen) was performed with the Human-7 Multiple Affinity Removal Spin (MARS) cartridge from Agilent Technologies following manufacturer’s protocol. Thirty (30) µg of depleted protein were reduced with 4 mM 1.4-Dithiothreitol for 1h at 37°C and alkylated with 8 mM iodoacetamide for 30 min at 25°C in the dark. Afterwards, samples were overnight digested (pH 8.0, 37°C) with sequencing-grade Trypsin/Lys-C Protease Mix (ThermoFisher Scientific, CA, USA) at enzyme:protein ratio of 1:50. Digestion was quenched by acidification with 1% (v/v) formic acid and peptides were desalted on Oasis HLB SPE column (Waters, Massachusetts, USA) before TMT 11-plex labeling (Thermo Fisher Scientific, CA, USA) following manufacturer instructions. To normalize all samples in the study along the different TMT-multiplexed batches used, a pool containing all the samples was labeled with TMT-126 tag and included in each TMT The different TMT 11-plex batches were desalted on Oasis HLB SPE columns before the nanoLC-MS analysis. Labelled and multiplexed peptides were loaded on a trap nano-column (100 μm I.D.; 2cm length; 5μm particle diameter, ThermoFisher Scientific, CA, USA) and separated onto a C18 reversed phase (RP) nano-column (75μm I.D.; 15cm length; 3μm particle diameter, Nikkyo Technos Co. LTD, Japan) on an EASY-II nanoLC from Thermo Fisher. The chromatographic separation was performed with a 180 minutes (min) gradient using Milli-Q water (0.1% formic acid) and acetonitrile (0.1% formic acid) as mobile phase at a flow rate of 300 nL/min. Each TMT-plex was analysed twice to increase the peptide and protein coverage. Mass spectrometry analyses were performed on an LTQ-Orbitrap Velos Pro from ThermoFisher by an enhanced FT-resolution MS spectrum (R=30,000 FHMW) followed by a data dependent FT-MS/MS acquisition (R=15,000 FHMW, 40% HCD) from the most intense ten parent ions with a charge state rejection of one and dynamic exclusion of 0.5 minutes. Protein identification and quantification was performed on Proteome Discoverer software v.1.4.0.288 (ThermoFisher Scientific, CA, USA) by Multidimensional Protein Identification Technology (MudPIT) combining the two raw data files obtained from each sample. For protein identification, all MS and MS/MS spectra were analyzed using Mascot search engine (v.2.5) combing Homo sapiens (74449 entries) and contaminants (247 entries) databases. Two missed cleavages were allowed and an error of 0.02 Da for FT-MS/MS fragmentation mass and 10.0 ppm for a FT-MS parent ion mass were allowed. TMT-10plex was set as quantification modification and oxidation of methionine and acetylation of N-termini were set as dynamic modifications, whereas carbamidomethylation of cysteine was set as static modifications. The false discovery rate (FDR) and protein probabilities were calculated by Percolator. For protein quantification, the ratios between each TMT-label against 126-TMT label were used and quantification results were normalized based on protein median.

***Lipidomic analysis***

For the extraction of hydrophobic lipids, a liquid-liquid extraction with chloroform:methanol (2:1) based on Folch procedure was performed by adding four volumes of chloroform:methanol (2:1) containing internal standard mixture (Lipidomic SPLASH®) to serum (20 µL). Then, the samples were mixed and incubated at -20°C for 30 min. Afterwards, water with NaCl (0.8 %) was added and mixture was centrifuged at 15,000 rpm. Lower phase was recovered, evaporated to dryness and reconstituted with methanol:methyl-tert-butyl ether (9:1). Samples were analyzed on a 1290 Infinity UHPLC coupled to a 6550 qTOF mass spectrometer from Agilent Technologies (Santa Clara, CA, USA). The chromatographic separation consists in an elution with a ternary mobile phase containing water, methanol and 2-propanol with 10mM ammonium formate and 0.1% formic acid. The stationary phase was a C18 column (Kinetex EVO C18 Column, 2.6 µm, 2.1 mm X 100 mm) that allows the sequential elution of the more hydrophobic lipids such as lysophospholipids, sphingomyelins, phospholipids, diglycerides, cholesteryl esters and triglycerides, among others. The identification of lipid species was performed by matching their accurate mass and tandem mass spectrum, when available, to Metlin-PCDL from Agilent containing more than 40,000 metabolites and lipids. Chromatographic behavior of pure standards for each family and bibliographic information was used to ensure their putative identification. After putative identification of lipids, these were semi quantified in terms of internal standard response ratio using one internal standard for each lipid family.

***Metabolomic analysis***

For metabolomics analysis, a protein precipitation extraction was performed by adding eight volumes of methanol:water (8:2) containing internal standard mixture (succinic acid-d4, myristic acid-d27, glycerol-13C3 and D-glucose-13C6) to serum samples. Samples were mixed and incubated at 4°C for 10 minutes, centrifuged at 15,000 rpm and supernatant was evaporated to dryness before compound derivatization (methoxyamine hydrochloride and MSTFA +1% TMCS). Samples were analyzed on a 7200 GC-qTOF from Agilent Technologies (Santa Clara, CA, USA). The chromatographic separation was based on Fiehn Method, using a J&W Scientific HP5-MS (30 m x 0.25 mm i.d., 0.25 µm film capillary column and helium as carrier gas using an oven program from 60 to 325°C. Ionization was done by electronic impact (EI), with electron energy of 70eV and operated in full scan mode, recording data in a range between 35 and 700 m/z at a scan rate of 5 spec/s. Targeted compounds from central carbon metabolism were identified using pure standards, in addition, a screening for the identification of more metabolites was performed by matching their EI mass spectrum and retention time to metabolomic Fiehn library (from Agilent) which contains more than 1.400 metabolites. After putative identification of metabolites, these were semi-quantified in terms of internal standard response ratio.

**Statistical analysis**

Statistical significance regarding the distribution of qualitative variables such as gender, symptoms, comorbidities, oxygen and clinical treatments was determined using Chi-squared test. Global significance regardless the disease severity was calculated using Chi-squared for qualitative and Kruskal-Wallis test for quantitative variables. ANOVA test followed by post-hoc Bonferroni correction was employed to assess significant differences between groups of severity in the data generated by the multi-omics analysis and blood biochemical parameters. Correlation analyses were evaluated using the Spearman rank test including the top selected proteins, metabolites and lipids with the highest classification accuracy (Random forest analyses). Statistical analyses were performed using SPSS (version 21.0, SPSS Inc., Chicago, IL), and graphical representations were generated with GraphPad Prism software (version 9.0, GraphPad Inc., San Diego, CA), Metaboanalyst 5.0 and String software. Random forest analyses were performed to determine the proteins, lipids and metabolites with higher accuracy in classifying patients according to disease severity. Pathway enrichment analyses were conducted to explore the association of the selected biomarkers and COVID-19 severity. Random forest and pathway enrichment analyses were both done using Metaboanalyst 5.0 software. Binary logistic regression models and Receiver operating characteristic (ROC) curves were generated by SPSS (version 21.0, SPSS Inc., Chicago, IL) to evaluate the potential accuracy of the selected biomarkers for predicting COVID-19 severity. Logistic regression analyses were performed in a randomly selected subset of patients (50% from mild group and 50% from critical group) and, to optimize all the model components, the stepwise variable selection was utilized. The results were considered statistically significant at P < 0.05.

**RESULTS AND DISCUSSION**

**Characteristics of the patient cohort**

In our COVID-19 cohort median age increased with severity, whereas female sex predominated in the mild group. In contrast to severe and critical patients, patients with mild cases rarely suffered from comorbidities (Figure 1Ba, Table S1). Hypertension, which was previously described in the literature as the strongest risk factor associated with disease severity (Chen et al., 2020), was the most common comorbidity in all groups, and was more predominant in severe and critical patients. Diabetes mellitus and obesity were also present at significantly higher levels in the severe and critical groups compared to the mild group. Hence, according to previous studies [11], individuals presenting comorbidities such as hypertension, obesity, diabetes and cardiovascular diseases may have a worse prognosis and are at higher risk of suffering from severe respiratory distress with subsequent oxygen and drug requirements.

Regarding the symptoms reported at the acute infection, anosmia was reported more frequently by patients in the mild group, and the incidence of dyspnea was significantly higher in the critical group (Figure 1Bb). Drug administration and the need for oxygen and aggressive treatments were consistent with the severity of the disease (Figure 1Bc and 1Bd). Thus, medications administered to patients in the mild group included antibiotics such as azithromycin, corticosteroids were required by some patients in the severe and critical groups (Figure 1Bc), and nearly all patients in the critical group required hydroxychloroquine and lopinavir/ritonavir. Low-flow oxygen therapies were mainly necessary for severe patients, some of whom required high-flow oxygen administration (Figure 1Bd). Among patients suffering a severe disease, 38.7% were intubated, and approximately half required vasopressor administration or dialysis (Figure 1Bd). Notably, two patients in the severe group were receiving dialysis before SARS-CoV-2 infection. Twenty-eight patients died from COVID-19, 8 of whom belonged to the severe group and 17 to the critical group.

**Table S1. Demographic and clinical features of COVID-19 study cohort.**

| Variables | COVID-19 group | | | |
| --- | --- | --- | --- | --- |
|  | **Mild (n=77)** | **Severe (n=134)** | **Critical (n=62)** | **P value** |
| Male | 26 (33.8) | 74 (55.2) | 42 (67.7) | n.s. |
| Age, years | 41.0 (28.0-53.0) | 64.0 (49.8-75.0) | 65.0 (53.0-74.25) | <0.0001 |
| Comorbidities – no. (%) | | | | |
| Obesity | 6 (7.8) | 23 (17.2) | 12 (19.4) | <0.0001 |
| Metabolic syndrome | 0 (0) | 9 (6.7) | 8 (12.9) | <0.0001 |
| Diabetes mellitus | 2 (2.6) | 24 (17.9) | 18 (29.0) | <0.0001 |
| Hypertension | 7 (9.1) | 60 (44.8) | 30 (48.4) | <0.0001 |
| Cardiovascular disease | 4 (5.2) | 31 (23.1) | 6 (9.7) | <0.0001 |
| COPD | 2 (2.6) | 4 (3.0) | 4 (6.5) | <0.0001 |
| Cancer | 2 (2.6) | 9 (6.7) | 5 (8.1) | <0.0001 |
| HIV | 0 (0) | 2 (1.5) | 1 (1.6) | <0.0001 |
| COVID-19 Symptoms | | | | |
| Fever | 48 (62.3) | 69 (51.5) | 41 (66.1) | 0.006 |
| Cough | 49 (63.6) | 89 (66.4) | 43 (69.4) | <0.0001 |
| Fatigue | 21 (27.3) | 0 (0) | 0 (0) | <0.0001 |
| Dyspnea | 0(0) | 62 (46.3) | 35 (56.5) | <0.0001 |
| Anosmia | 26 (33.8) | 10 (7.5) | 3 (4.8) | <0.0001 |
| Oxygen therapy and intensive care | | | | |
| Oxygen required | 0(0) | 95 (70.9) | 62(100) | <0.0001 |
| Low-flow oxygen administration  (Ventimask or nasal prongs) | 0(0) | 85 (63.4) | 2 (3.2) | <0.0001 |
| High-flow oxygen administration/NIMV | 0(0) | 8 (6.0) | 6 (9.7) | 0.031 |
| MV/intubation | 0(0) | 0(0) | 24 (38.7) | <0.0001 |
| Vasopressors or dialysis required | 0(0) | 2 (1.5) | 30 (48.4) | <0.0001 |
| Mortality | | | | |
| Exitus | 0 (0) | 8 (6.0) | 17 (27.4) | <0.0001 |
| Medicines subscribed | | | | |
| Hydroxychloroquine | 29 (37.7) | 110 (82.1) | 58 (93.5) | <0.0001 |
| Azithromycin | 8 (10.4) | 33 (24.6) | 14 (22.6) | <0.0001 |
| Lopinavir/ritonavir | 20 (26.0) | 90 (67.2) | 56 (90.3) | <0.0001 |
| Tocilizumab | 0 (0) | 2 (1.5) | 7 (11.3) | <0.0001 |
| Interferon | 0 (0) | 2 (1.5) | 13 (21.0) | <0.0001 |
| Corticosteroids | 0 (0) | 29 (21.6) | 24 (38.7) | <0.0001 |
| Remdesivir | 0 (0) | 3 (2.2) | 0 (0) | <0.0001 |

Data are presented as n (%) or median (interquartile range: 25-75). P values comparing mild, severe and critical patients were computed using non-parametric Kruskal-Wallis test for continuous data and χ2 test for categorical data. P value < 0.05 was considered significant. COPD, chronic obstructive pulmonary disease; NIMV, non-invasive mechanical ventilation; MV, mechanical ventilation.

The serum biochemical composition was also characterized at the time of admission in the whole cohort, including both routine and inflammatory parameters (Figure S1).

**Figure S1**. **Biochemical profile of COVID-19 study cohort**. Y axis represent blood levels of biochemical parameters expressed as median and CI 95%. Double dashed lines show the recommended interval of each parameter for healthy subjects and one dashed line indicates normal level below the line. (*) denotes significant p-values (<0.05) obtained by ANOVA test followed by post-hoc Bonferroni correction for mean biochemical values compared between mild, severe and critical COVID-19 groups of patients.

The most significant differences were observed between mild and critical patients, revealing a well-differentiated blood pattern for severe disease. Thus, critically ill patients with COVID-19 exhibited higher glucose concentrations, leukocytosis, neutrophilia, and lymphopenia; reduced levels of platelets and monocytes; and significantly higher serum concentrations of fibrinogen, IL-6, and C-reactive protein (CRP). Elevated levels of fibrinogen, IL-6, and CRP were observed in all groups compared to optimal values for healthy subjects, with a significant increase observed in the most severe cases, a feature previously reported in other similar studies [12]. Indeed, fibrinogen, IL-6 and CRP are signs of an inflammatory response and are widely associated with adverse COVID-19 outcomes [13].

**Proteins from complement and coagulation cascades are key factors related to a critical COVID-19 progression**

The proteomics analysis identified 240 proteins, 65 of which revealed significant increases (33 proteins) or decreases (32 proteins) in abundance that were correlated with disease severity (Fig. 2A, B). A pathway enrichment analysis was then conducted to explore the association between the significantly up- or downregulated proteins and COVID-19 severity. Overall, the interconnected up- and downregulated genes were enriched in 77 pathways, and the 10 relevant pathways whose impact values were greater than 0.1 (p < 0.05) were further considered (Fig. 2C). Interestingly, complement and coagulation cascades were by far the most significantly enriched biological pathways (impact value of 0.44 and *p value* of 3.22·10^-31^). In fact, most of the protein-coding genes present at higher abundance in critically ill patients with COVID-19 were involved in the complement and coagulation cascade pathways (C1S, C9, C3/C5 convertase, C5, C1RL, F9, VNT, C4BPA, C1QC, CFI and C1R). Notably, coagulopathy is a drastic phenomenon occurring in a high proportion of critically ill patients with COVID-19 and is specifically related to COVID-19 mortality. In addition, other protein-coding genes not directly related to the complement or coagulation cascade, such as carboxypeptidases (CPB2), protease inhibitors (SERPINF1, SERPINA3, SFMBT2 and SERPING1), acute phase proteins (LBP, CRP, SAA1 and SAA2), extracellular matrix stabilizers (ITIH3 and ITIH4) and antimicrobial enzymes (DEFA1, LRG1, LYZ, and ORM1), were also significantly perturbed in severely and critically ill patients. The increased expression of these proteins showed interconnected functions (ECM-interaction receptor, focal adhesion, regulation of actin cytoskeleton and bacterial infection) (Fig. 2B, upper panel). Concretely, abnormal upregulation of acute phase proteins (AAPs) released by the liver into circulation to reduce and modulate the immune response in case of infection or tissue damage promotes excess inflammation in patients with viral pneumonia (Gelain and Bonsembiante, 2019; Perez, 2019) and might be responsible for the aggravation of severe and critical COVID-19 cases in patients. On the other hand, endothelial dysfunction was indicated by a significant increase in the levels of extracellular matrix-remodeling proteins and antimicrobial enzymes in critical patients compared with mild and severe patients. These molecule levels, if altered, provide an opportunity for the virus to evade host immune responses and increase the possibility of concomitant secondary bacterial infection along with an exacerbation of the immune response.

Conversely, the 32 proteins with reduced abundance in critically ill patients with COVID-19 were mostly associated with lipid transport (apolipoproteins; APOs). The dysfunction of lipid transport was reflected by the decreased levels of APOA1, APOB, APOC4, APOC4-APOC2, APOD, APOL and APOM observed in severely and critically ill patients compared to patients with mild cases (Fig. 2B, bottom panel). Apolipoproteins transport lipids (e.g., cholesterol) in blood, lymph and cerebrospinal fluid, acting as [structural](https://en.wikipedia.org/wiki/Secondary_structure) components of lipoprotein particles. Lipids, which are important structural components of cellular membranes, play a central role in viral infection processes, participating in the regulation of transmembrane molecule trafficking, virus attachment and endocytosis [16]. Indeed, scientific evidence supports the fact that hepatitis C virus (HCV) benefits from host apolipoproteins, enabling viral entrance, assembly, and transmission into the host system [17].

Finally, a random forest analysis was performed to determine the accuracy of the altered proteins in classifying patients according to disease severity (Figure 2D). The analysis identified fetuin-A (AHSG) and inter-α-trypsin inhibitor 3 (ITIH3) as the protein-coding genes with the highest accuracy to distinguish between mild and critical cases. On the one hand, significantly higher circulating AHSG concentrations were detected in patients with a mild illness than in critically ill patients with COVID-19 (Fig. 2A). Accordingly, the glycoprotein AHSG has previously been reported to be downregulated during the acute phase response in patients on dialysis, and its deficiency is linked to inflammation and is responsible for cardiovascular mortality [18]. On the other hand, ITIH3, which was increased in severely and critically ill patients compared to patients with a mild illness (Fig. 2A), was recently reported in a study performed on a COVID-19 cohort comparing survivors and nonsurvivors [19], in which a biomarker panel included both ITIH3 and AHSG as markers of mortality in patients with COVID-19.

Therefore, our study confirmed previous results that appointed the complement system, coagulation cascade, acute phase/inflammation and extracellular matrix remodeling/fibrosis as pathways directly implicated in COVID-19 physiopathology and responsible for disease progression [20–25]. Interestingly, our data also reported the alteration in the circulating concentrations of certain proteins involved in coagulation cascades, cell adhesion, and lipid transport that might be determinants of COVID-19 progression, which have not been previously reported in the other proteomic studies [26].

**
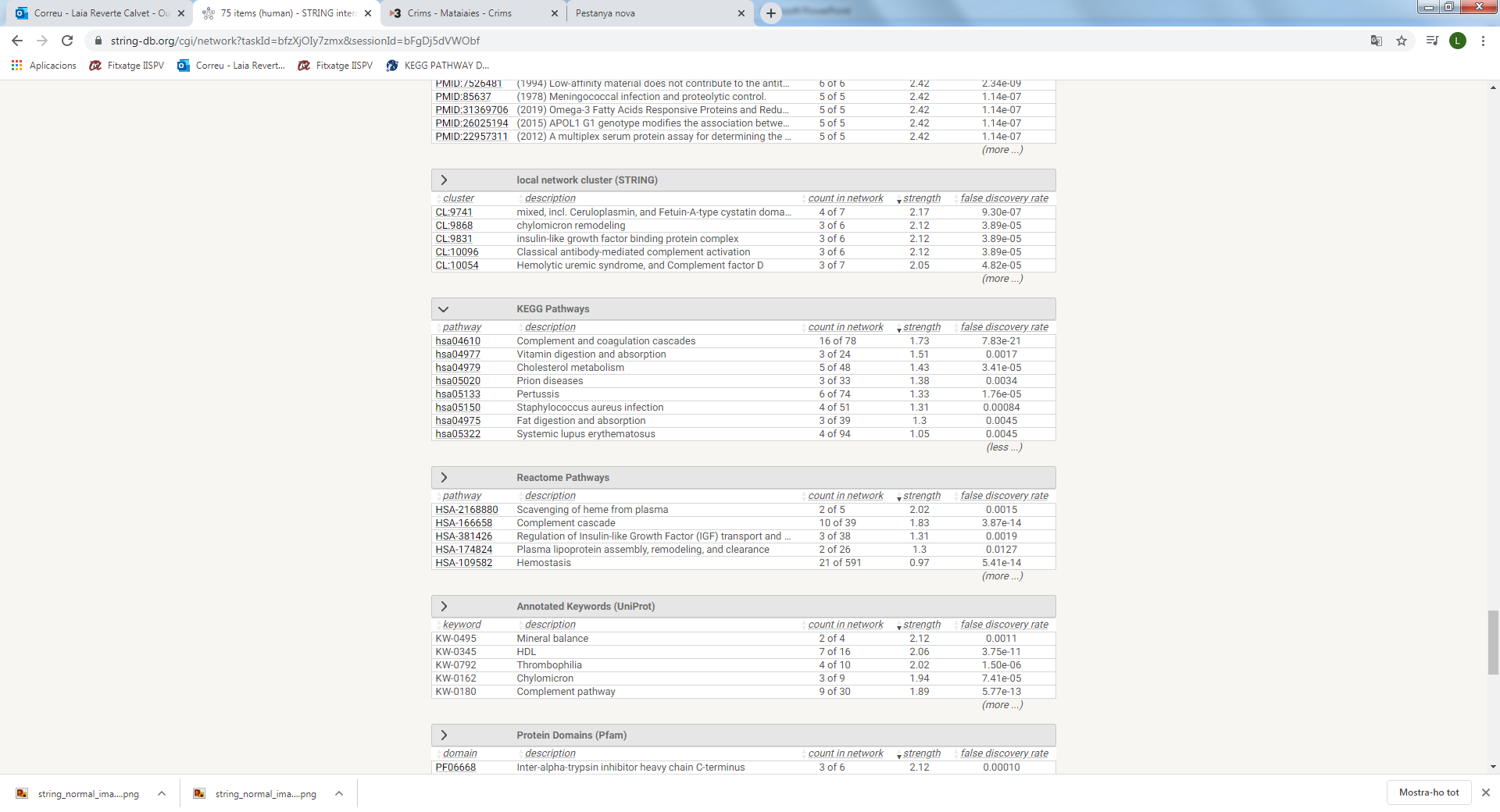
Glutamic acid and ChoE (18:0) as predictive biomarkers of the most severe COVID-19 outcomes**Seventy-four metabolites and 112 lipids were analyzed and quantified, and the significant differences between the three groups are presented in a heatmap as mean values of the normalized relative abundance (Figure 3A). Among the 74 metabolites, our study identified 29 metabolites that were significantly increased with severity (Fig. 3A, from red to green) and 5 metabolites that were significantly decreased in patients with worse clinical progression (Figure 3A, from green to red). On the other hand, the lipidomic analysis revealed 18 lipids that basically included the glycerolipid family and were present at increased abundance in severely and critically ill patients compared with mild cases (Figure 3A, from red to green) and 10 lipids that were predominantly from cholesterol ester and sphingomyelin families with significantly lower abundance in the severe and critical groups compared to the mild group (Figure 4A, from green to red).

Interestingly, among the groups of metabolites whose levels increased with severity, we identified some intermediaries of amino acid catabolism (2-hydroxyisobutyric acid, 2-hydroxybutyric acid, 3-hydroxybutyric acid, 2-hydroxyisovaleric acid and 3-hydroxyisovaleric acid) and phenylalanine; both pathways are strictly associated with an important role in cell homeostasis and immune system responses. On the one hand, an increase in α-hydroxyl amino acid levels indicates enhanced amino acid catabolism. On the other hand, plasma concentrations of phenylalanine increase upon inflammation and an excess immune activation (with release of IFNs and TNF-α) induced by infections, cancer or autoimmune diseases. In addition, the upregulation of phenylalanine metabolism is associated with neuropsychiatric symptoms such as mood changes, depression and fatigue [27]. Moreover, many of the altered metabolites that were either up- or downregulated were amino acids and/or sugars involved in central carbon metabolism (oxalic acid, D-gluconic acid, urea, octanoic acid, D-mannitol, D-mannonic acid, 4-hydroxyphenyllactic acid, galacturonic acid, glutamic acid, citric acid, aspartic acid and urea). Carbon metabolism consists of a biochemical pathway that connects carbohydrates, [fat](https://en.wikipedia.org/wiki/Fat), and [protein](https://en.wikipedia.org/wiki/Protein) [metabolism](https://en.wikipedia.org/wiki/Metabolism) to generate the energy required for most essential biological life processes. In our cohort, the critically ill patients showed a significant increase in glucose and glutamic acid levels but a reduction in glutamine, citrate and uric acid levels compared to patients in both the mild and severe groups (Fig. 3A). These findings suggested that in response to a major glucose requirement induced by immune system activation due to SARS-CoV-2 infection, infected cells (lung epithelial cells, monocytes, nervous cells, etc.) shifted from anaerobic to aerobic glycolysis, a phenomenon known as the Warburg effect. This process, in turn, may prevent pyruvate from entering the TCA cycle and convert pyruvate to lactate, consistent with the observed increase in the levels of lactic acid derivatives such as 4-hydroxyphenyllactic acid in critically ill patients. Altogether, enhanced aerobic glycolysis along with mitochondrial dysfunction was depicted as an alternative cellular mechanism previously identified for cancer and other virus-infected cells [28]. The amino acid glutamate is a fundamental metabolite with a central role in multiple processes, including nitrogen assimilation, amino acid biosynthesis and signal transmission in neurons. Furthermore, glutamine and glutamate are both alternative carbon sources for energy production and amino acid and fatty acid biosynthesis. Viruses take advantage of the host cellular and metabolic machinery to ensure their optimal replication [29]. Based on our findings and recent *in vitro* studies [30], we reinforced the finding that SARS-CoV-2 seizes control of host glycolysis and glutaminolysis pathways to meet the requirements for carbon sources, leading to decreased mitochondrial functions and metabolic toxicity that are attributable to severe COVID-19 complications.

The lipidomic pattern observed herein similar to that reported in a recent study [31]. High TG levels are often a sign of other diseases, such as obesity and metabolic syndrome, a condition that increases the risks of heart disease and stroke. Consistent with the study by Shein and coworkers, critically ill patients with COVID-19 showed a reduced relative abundance of SMs and ChoEs compared to patients with a mild illness. SMs, cholesterol and its derivatives, as well as some phospholipids are important components of biomembranes, where they participate in signal transduction with a central role in modulating immune processes. Likewise, these lipids regulate cellular processes such as migration, adhesion, apoptosis and inflammation [32] and are known to induce macrophage activation, migration, apoptosis, etc. [33]. Given the strong evidence supporting the intimate connection between lipid metabolism and the immune system in acute inflammation induced by SARS-CoV-2, this specific lipidomic profile might be considered a marker of a poor prognosis.

Thus, a pathway enrichment analysis was conducted to further assess the functional implications of the differentially altered lipidomics and metabolomics patterns according to severity (Figure 3B). The selected compounds were enriched in 21 pathways and significantly enriched (p values<0.05) in 9 of these pathways, with the main enriched pathway being D-glutamine and D-glutamate metabolism (Figure 3B, left panel). As indicated above, glutamine and glutamate play central roles in controlling cell homeostasis but are also essential for virus replication, becoming the main source of carbon for amino acid and lipid biosynthesis, which might explain why this pathway obtained the highest enrichment score. Accordingly, the disturbed metabolic and lipidomic pathways were associated with 25 disorders (Fig. 3B, right panel), 13 of which were significantly enriched (p<0.05). Although the exact mechanism leading to seizures and epilepsy is not completely understood, recent studies proposed an increase in levels of excitatory neurotransmitters (such as glutamate), the presence of acute metabolic disorders, neuronal damage following infection and inflammation, fever, elevated cytokine levels or mitochondrial dysfunction, among others, as possible causes explaining these alterations and other neurological disorders arising from COVID-19 [34]. Therefore, all the data generated by the metabolomic and lipidomic blood analysis showed once again to what extent disturbances in essential metabolic, lipidomic and/or immunological mechanisms aggravate the disease, which may account for the unresolved SARS-CoV-2 infection that led to the typical complications reported for patients with the most critical COVID-19 outcomes.

A random forest analysis was then conducted to ascertain the power of the aforementioned modified metabolites and lipids as predictive biomarkers for COVID-19 evolution (Figure 3C). This analysis determined that the increase in the circulating glutamic acid concentration was the metabolite with the greatest potential to classify critically ill patients, and the reduction in ChoE (18:0) abundance was the lipid species with the strongest potential to identify individuals with a mild disease. Subsequently, prediction models were built to assess the predictive efficacy of these two compounds, and receiver operating characteristic (ROC) curves were constructed to evaluate their accuracy (Figure S2).

**Figure S2. Receiver operating characteristic (ROC) curves analysis**. ROC curves of glutamic acid and ChoE (18:0) alone and combined for distinguishing: **A)** mild from critical, **B)** mild from severe, and **C)** severe from critical COVID-19 patients**. D)** ROC curve analysis data obtained for the different pairwise comparisons, area under the curve (AUC) scores, significance, specificity and sensitivity.

ROC curves were generated alone and in combination through a binary logistic regression analysis comparing the mild vs. critical groups (Fig. S2A), mild vs. severe groups (Fig. S2B) and severe vs. critical groups (Fig. S2C). The combination of glutamic acid with ChoE (18:0) slightly improved the AUC values with respect to considering each compound independently (green lines, Fig. S2D), showing the highest accuracy to distinguish mild from critical illnesses, with an AUC of 0.963 and a specificity and a sensitivity of 85.7 and 93.5%, respectively. These findings indicated the usefullness of glutamic acid and ChoE (18:9) as biomarkers to effectively discriminate between mild and critical COVID-19 and provide useful concepts for the rapid identification of clinical progression in critically ill patients during the early stage. In contrast, glutamic acid and ChoE (18:0) combined showed a poor capacity to differentiate between severely and critically ill patients (AUC<0.68, specificity<60% and sensitivity <66%) (Fig. S2C). This observation is consistent with the minor differences observed between severely and critically ill patients in the biochemical composition and metabolomic and lipidomic profiles. Therefore, to predict the progression from severe to critical illnesses more biomarkers should be taken into consideration. In line with our study, other authors proposed prognostic biomarkers panels based on the plasmatic metabolomics and lipidomics analyses of COVID-19 patients, some of them comparing controls with COVID-19-positive individuals [35, 36], and some others attempting to differenciate the degrees of disease severity [37–41].

**Glutamic acid, ChoE (18:0), fetuin-A and inter-α-trypsin inhibitor together increase the accuracy in differenciating mild from critically-ill patients with COVID-19**

The host response to SARS-CoV-2 infection is known to entail a complex set of immunological, genomic, metabolomic and lipidomic processes. Hence, we firmly believe that an understanding of the linkage or coregulation between the distinct classes of biomolecules might provide valuable insights into the pathophysiology of the disease. Hence, research in this direction is of particular interest for the most critical forms of COVID-19. With this purpose in mind, we performed an unsupervised Spearman correlation matrix analysis including the most enriched proteins (Fig S3A), metabolites and lipids (Fig S3B) determined by the Random forest analyses with significant differences according to the disease severity.

**Figure S3. Relative abundance of the most significantly enriched proteins (A), lipids and metabolites (B) according to disease severity, as determined by the random forest analyses.** For each group of COVID-19 patients, box plots show the mean relative abundance (in log scale for the lowest concentrations) with maximum (top box) and minimum (bottom box). Dots indicate individual values corresponding to each patient’s measure. Significant differences were evaluated (p-values (<0.05)) using ANOVA test followed by post-hoc Bonferroni correction for mean values comparing between mild, severe and critical COVID-19 groups of patients.

The heatmap identified four dense clusters of Spearman coefficients (Fig. 4A1-3). The first cluster, located in the upper left part of the heatmap, linked age to biochemical features, showing strong positive correlations with several of the selected biomolecules, particularly with those differentially altered in severe and critically-ill patients compared to patients with mild disease. On the one hand, the increase in blood CRP and neutrophil levels and the reduction in T-lymphocyte counts reflected the immune system overreaction induced by SARS-CoV-2 infection [42], which triggers cytokine release, as shown by the abundant IL-6 levels (Fig. S1). On the other hand, the reduced levels of platelets but higher levels of D-dimer and fibrinogen observed in critically ill patients accounted for the typical thrombocytopenia and the hypercoagulation state [43], which cause multiple organ damage and eventually the death of the subject. In relation to omics data, the heatmap revealed 3 relevant clusters within each omic molecule specifically marked by strong positive correlations for lipids (ρ=+0.7 to +1), weak positive correlations for metabolites and both positive and negative correlations within the group of protein-coding genes (ρ=+0.7 to +1 and -0.7 to -1, respectively). Notably, moderate correlations were identified between proteomics and metabolomics biomolecules, specially noticeable in the correlation matrix obtained for the mild and critically-ill group of patients (Fig 4A1 and 3, respectively). Although negative associations were more abundant in this cluster, some interesting positive correlations were also identified. Gelsolin (GSN) and citric acid were both detected in lower abundance in critical cases than in severe and mild cases (Fig. 2A and Fig. 3A, respectively). The downregulation of these two molecules is widely associated with coagulopathy and related to critical COVID-19 outcomes [44]. As expected, age also showed positive correlations with metabolites and amino acids that were more abundant in critically ill patients and involved in processes such as central carbon metabolism (glucose, glutamic acid, D-gluconic acid, D-mannitol, D-mannonic acid and oxalic acid), amino acid catabolism (2-hydroxybutyric acid and 3-hydroxyisovaleric acid) and phenylalanine metabolism (phenylalanine), which together disturbed the host’s metabolic state. Conversely, age was negatively correlated with APOA1 and APOM. Importantly, all these altered molecules related to carbon, amino acid and phenylalanine metabolism showing a positive correlation with age were negatively correlated with APOA1 and APOM. Therefore, these results showed the increasing potential of age as risk factor when combined with all the biomolecules responsible for the exacerbation of the immune response and alterations in coagulation, proteomic, lipidomic and metabolic processes. In addition, the disturbed proteomic, metabolomic and lipidomic patterns revealed in this study have been found to be associated with the progression and severity of COVID-19.

Finally, prediction models were built incorporating the omics biomolecules with the best classification accuracy to explore their predictive power as biomarkers for severe disease (Fig. 4B). The biomolecule with the best predictive capacity was AHSG, which slightly improved the sensitivity and AUC values (90.6% and 0.942, respectively) compared with those obtained with ChoE (18:0) (87.1% and 0.907, respectively) that was still appropriate but had a weaker predictive value. Notably, the combination of all the biomolecules (glutamic acid (GA), ChoE (18:0), AHSG and ITIH3) provided the highest accuracy in classifying patients with COVID-19 according to severity, significantly increasing the predictive capacity to discriminate mild from critical patients (AUC value of 0.994, 100% sensitivity and 85.9% specificity). Given that metabolomic and lipidomic biomarkers alone (Figure S2-C) were not effective enough to separate severe from critical cases, the power of all the selected biomarkers (Figure2D-Figure3C) in the separation of the patients with most adverse outcomes was tested through a ROC curve analysis (Figure 4C, AUC=0.811). Finally, to validate the accuracy of the four selected biomarkers to properly predict mild and critical outcomes, a regression model was performed in a randomly selected set of patients (50% of patients from each group). The results obtained confirmed the sutability of fetuin-A, inter-α-trypsin inhibitor, glutamic acid and ChoE (18:0) in distinguishing mild from critical outcomes (Figure 4D).

**LIMITATIONS**

Considering that COVID-19 is a novel emerging disease, several limitations are worthy to be mentioned. The categorization based on COVID-19 severity to perform subgroup analyses led to misclassification bias since it does not take into account other patients characteristics such as pre-existing comorbidities, nutrition habits and medication administered before the blood sample collection that, in turn, could have a special effect on the metabolomics and lipidomics readouts. However, a direct consequence of performing multiple underpowered subgroup comparisons is that readers can be misled into believing that results provide reliable evidence that differences among groups have similar effects in all patients, which could potentially mask important subgroup effects owing to inadequate power. For that reason and considering that subgroups analysis will include less than 25% of the subjects categorized in each group of severity and that the main objective of the study was to evaluate COVID-19 severity progression regardless of the previous patients' health status, we discard performing multiple subgroup comparisons other than those related to COVID-19 progression. Another limitation is the sample size to firmly establish the proposed molecules as predictive biomarkers of critical COVID-19 outcomes, but the fact that the cohort is composed of more than two hundred patients from 3 hospitals from different locations makes it heterogeneous and representative of the Spanish population. Thus, although the modelling regression analysis tested the power of the selected biomarkers in a randomly selected set of patients, validation studies in larger cohorts are still needed to establish the combination of fetuin-A, inter-α-trypsin inhibitor, glutamic acid and ChoE (18:0) as predictive biomarkers of critical COVID-19 outcomes.

**References**

1. Baden LR, Rubin EJ (2020) Covid-19 — The Search for Effective Therapy. N Engl J Med 382:1851–1852. https://doi.org/10.1056/nejme2005477

2. Yang X, Yu Y, Xu J, et al (2020) Clinical course and outcomes of critically ill patients with SARS-CoV-2 pneumonia in Wuhan, China: a single-centered, retrospective, observational study. Lancet Respir Med 8:475–481. https://doi.org/10.1016/S2213-2600(20)30079-5

3. Scialpi M, Scialpi S, Piscioli I, et al (2020) Pulmonary thromboembolism in critical ill COVID-19 patients. Int. J. Infect. Dis. 95:361–362

4. Kermali M, Khalsa RK, Pillai K, et al (2020) The role of biomarkers in diagnosis of COVID-19 – A systematic review. Life Sci. 254

5. Rana R, Rathi V, Ganguly NK (2020) A comprehensive overview of proteomics approach for COVID 19: new perspectives in target therapy strategies. J Proteins Proteomics 11:223–232. https://doi.org/10.1007/s42485-020-00052-9

6. Ray S, Srivastava S (2020) COVID-19 Pandemic: Hopes from Proteomics and Multiomics Research. Omi A J Integr Biol 24:457–459. https://doi.org/10.1089/omi.2020.0073

7. Lin B, Liu J, Liu Y, Qin X (2021) Progress in understanding COVID-19: insights from the omics approach. Crit. Rev. Clin. Lab. Sci. 58:242–252

8. Wang G-Q, Zhao L, Wang X, et al (2021) Diagnosis and Treatment Protocol for COVID-19 Patients (Tentative 8th Edition): Interpretation of Updated Key Points. Infect Dis Immun 1:17. https://doi.org/10.1097/ID9.0000000000000002

9. Li J, Huang DQ, Zou B, et al (2021) Epidemiology of COVID-19: A systematic review and meta-analysis of clinical characteristics, risk factors, and outcomes. J Med Virol 93:1449–1458. https://doi.org/10.1002/jmv.26424

10. Chen Y, Zheng Y, Yu Y, et al (2020) Blood molecular markers associated with COVID‐19 immunopathology and multi‐organ damage. EMBO J 39:. https://doi.org/10.15252/EMBJ.2020105896

11. Perpiñan C, Bertran L, Terra X, et al (2021) Predictive biomarkers of COVID-19 severity in SARS-CoV-2 infected patients with obesity and metabolic syndrome. J Pers Med 11:227. https://doi.org/10.3390/jpm11030227

12. Mudatsir M, Fajar JK, Wulandari L, et al (2021) Predictors of COVID-19 severity: a systematic review and meta-analysis. F1000Research 9:1107. https://doi.org/10.12688/f1000research.26186.2

13. Qin C, Zhou L, Hu Z, et al (2020) Dysregulation of immune response in patients with coronavirus 2019 (COVID-19) in Wuhan, China. Clin Infect Dis 71:762–768. https://doi.org/10.1093/cid/ciaa248

14. Perez L (2019) Acute phase protein response to viral infection and vaccination. Arch. Biochem. Biophys. 671:196–202

15. Elena Gelain M, Bonsembiante F (2019) Acute phase proteins in marine mammals: State of art, perspectives and challenges. Front. Immunol. 10

16. Abu-Farha M, Thanaraj TA, Qaddoumi MG, et al (2020) The role of lipid metabolism in COVID-19 virus infection and as a drug target. Int. J. Mol. Sci. 21

17. Wrensch F, Crouchet E, Ligat G, et al (2018) Hepatitis C virus (HCV)-apolipoprotein interactions and immune evasion and their impact on HCV vaccine design. Front. Immunol. 9

18. Ketteler M, Bongartz P, Westenfeld R, et al (2003) Association of low fetuin-A (AHSG) concentrations in serum with cardiovascular mortality in patients on dialysis: A cross-sectional study. Lancet 361:827–833. https://doi.org/10.1016/S0140-6736(03)12710-9

19. Völlmy F, van den Toorn H, Chiozzi RZ, et al (2021) A serum proteome signature to predict mortality in severe covid-19 patients. Life Sci Alliance 4:. https://doi.org/10.26508/LSA.202101099

20. D’Alessandro A, Thomas T, Dzieciatkowska M, et al (2020) Serum Proteomics in COVID-19 Patients: Altered Coagulation and Complement Status as a Function of IL-6 Level. J Proteome Res 19:4417–4427. https://doi.org/10.1021/acs.jproteome.0c00365

21. Messner CB, Demichev V, Wendisch D, et al (2020) Ultra-High-Throughput Clinical Proteomics Reveals Classifiers of COVID-19 Infection. Cell Syst 11:11-24.e4. https://doi.org/10.1016/j.cels.2020.05.012

22. Park J, Kim H, Kim SY, et al (2020) In-depth blood proteome profiling analysis revealed distinct functional characteristics of plasma proteins between severe and non-severe COVID-19 patients. Sci Rep 10:. https://doi.org/10.1038/s41598-020-80120-8

23. Su Y, Chen D, Yuan D, et al (2020) Multi-Omics Resolves a Sharp Disease-State Shift between Mild and Moderate COVID-19. Cell 183:1479-1495.e20. https://doi.org/10.1016/j.cell.2020.10.037

24. Shu T, Ning W, Wu D, et al (2020) Plasma Proteomics Identify Biomarkers and Pathogenesis of COVID-19. Immunity 53:1108-1122.e5. https://doi.org/10.1016/j.immuni.2020.10.008

25. Memon D, Barrio‐Hernandez I, Beltrao P (2021) Individual COVID-19 disease trajectories revealed by plasma proteomics. EMBO Mol Med 13:. https://doi.org/10.15252/EMMM.202114532

26. McArdle A, Washington KE, Chazarin Orgel B, et al (2021) Discovery Proteomicsfor COVID-19: Where We Are Now. J Proteome Res 20:4627. https://doi.org/10.1021/ACS.JPROTEOME.1C00475

27. Strasser B, Sperner-Unterweger B, Fuchs D, Gostner JM (2017) Mechanisms of inflammation-associated depression: Immune influences on tryptophan and phenylalanine metabolisms. In: Current Topics in Behavioral Neurosciences. Curr Top Behav Neurosci

28. Dyer A, Schoeps B, Frost S, et al (2019) Antagonism of glycolysis and reductive carboxylation of glutamine potentiates activity of oncolytic adenoviruses in cancer cells. Cancer Res 79:331–345. https://doi.org/10.1158/0008-5472.CAN-18-1326

29. Thaker SK, Ch’ng J, Christofk HR (2019) Viral hijacking of cellular metabolism. BMC Biol. 17:59

30. Krishnan S, Nordqvist H, Ambikan AT, et al (2021) Implications of central carbon metabolism in SARS-CoV-2 replication and disease severity. bioRxiv 2021.02.24.432759. https://doi.org/10.1101/2021.02.24.432759

31. Masana L, Correig E, Ibarretxe D, et al (2021) Low HDL and high triglycerides predict COVID-19 severity. Sci Rep 11:. https://doi.org/10.1038/s41598-021-86747-5

32. Hannun YA, Obeid LM (2018) Sphingolipids and their metabolism in physiology and disease. Nat. Rev. Mol. Cell Biol. 19:175–191

33. Weigert A, Weis N, Brüne B (2009) Regulation of macrophage function by sphingosine-1-phosphate. Immunobiology 214:748–760

34. Nikbakht F, Mohammadkhanizadeh A, Mohammadi E (2020) How does the COVID-19 cause seizure and epilepsy in patients? The potential mechanisms. Mult. Scler. Relat. Disord. 46

35. Meoni G, Ghini V, Maggi L, et al (2021) Metabolomic/lipidomic profiling of COVID-19 and individual response to tocilizumab. PLOS Pathog 17:e1009243. https://doi.org/10.1371/JOURNAL.PPAT.1009243

36. Barberis E, Timo S, Amede E, et al (2020) Large-Scale Plasma Analysis Revealed New Mechanisms and Molecules Associated with the Host Response to SARS-CoV-2. Int J Mol Sci 21:1–25. https://doi.org/10.3390/IJMS21228623

37. López-Hernández Y, Monárrez-Espino J, Oostdam ASH van, et al (2021) Targeted metabolomics identifies high performing diagnostic and prognostic biomarkers for COVID-19. Sci Rep 11:. https://doi.org/10.1038/S41598-021-94171-Y

38. Danlos FX, Grajeda-Iglesias C, Durand S, et al (2021) Metabolomic analyses of COVID-19 patients unravel stage-dependent and prognostic biomarkers. Cell Death Dis 12:. https://doi.org/10.1038/S41419-021-03540-Y

39. Shen B, Yi X, Sun Y, et al (2020) Proteomic and Metabolomic Characterization of COVID-19 Patient Sera. Cell 182:59-72.e15. https://doi.org/10.1016/j.cell.2020.05.032

40. Song JW, Lam SM, Fan X, et al (2020) Omics-Driven Systems Interrogation of Metabolic Dysregulation in COVID-19 Pathogenesis. Cell Metab 32:188-202.e5. https://doi.org/10.1016/J.CMET.2020.06.016

41. Wu D, Shu T, Yang X, et al (2020) Plasma metabolomic and lipidomic alterations associated with COVID-19. Natl Sci Rev 7:1157–1168. https://doi.org/10.1093/NSR/NWAA086

42. Cao W, Li T (2020) COVID-19: towards understanding of pathogenesis. Cell Res 30:367–369. https://doi.org/10.1038/s41422-020-0327-4

43. Kuchi Bhotla H, Kaul T, Balasubramanian B, et al (2020) Platelets to surrogate lung inflammation in COVID-19 patients. Med. Hypotheses 143

44. Overmyer KA, Shishkova E, Miller IJ, et al (2021) Large-Scale Multi-omic Analysis of COVID-19 Severity. Cell Syst 12:23-40.e7. https://doi.org/10.1016/j.cels.2020.10.003
